# Infrared Thermographic Evaluation Following Hemilaminectomy in Dogs with Thoracolumbar Intervertebral Disc Extrusion: A Pilot Study

**DOI:** 10.3390/ani16121796

**Published:** 2026-06-10

**Authors:** Cristian Zaha, Larisa Schuszler, Liliana Cărpinișan, Alina Ghișe, Tudor Căsălean, Văduva Cristina, Bogdan Sicoe, Ciprian Rujescu, Florin Vlad, Janos Degi, Roxana Dascălu

**Affiliations:** 1 Horia Cernescu Research Unit, Faculty of Veterinary Medicine, University of Life Sciences “Regele Mihai I” from Romania, 300645 Timisoara, Romania; cristian.zaha@usvt.ro (C.Z.); larisaschuszler@usvt.ro (L.S.); lilianacarpinisan@usvt.ro (L.C.); alinaghise@usvt.ro (A.G.); tudor.casalean@usvt.ro (T.C.); cristina.vaduva@usvt.ro (V.C.); bogdan.sicoe@usvt.ro (B.S.); florin.vlad@usvt.ro (F.V.); janosdegi@usvt.ro (J.D.); 2 Management and Rural Development Department, Faculty of Management and Rural Tourism, University of Life Sciences “Regele Mihai I” from Romania, 300645 Timisoara, Romania; rujescu@usvt.ro

**Keywords:** intervertebral disc extrusion, hemilaminectomy, infrared thermography, wound healing, thoracolumbar spine

## Abstract

(1) Background: Intervertebral Disc Disease (IVDD) represents one of the most frequently encountered neurological disorders in canine patients and often necessitates surgical decompression by means of hemilaminectomy. Objective methods for evaluating postoperative inflammatory response and tissue healing remain limited. This study aimed to evaluate temporal changes in local cutaneous temperature and thermal patterns in dogs undergoing hemilaminectomy for thoracolumbar intervertebral disc extrusion using Infrared Thermography; (2) Methods: Fifteen dogs diagnosed with Hansen type I thoracolumbar intervertebral disc extrusion were included. Diagnosis was established through neurological examination and computed tomography. Thermographic assessment of the thoracolumbar region (T11–L3) was performed preoperatively (Day 0), 24 h postoperatively (Day 1), and 7 days after surgery (Day 7) using an FLIR E50 thermal camera. Mean cutaneous temperature and thermal distribution patterns were analyzed. Statistical evaluation was performed using Repeated Measures ANOVA and the Friedman test; (3) Results: Significant temporal differences in local cutaneous temperature were identified between the evaluated time points (*p* < 0.001). Thermographic assessment demonstrated progressive modifications in thermal distribution throughout the postoperative period. No postoperative complications, including seroma formation, wound dehiscence, or fistula development, were observed; (4) Conclusions: Infrared thermography enabled identification of significant postoperative thermal changes following hemilaminectomy and may represent a useful complementary non-invasive method for postoperative monitoring of tissue healing in dogs.

## Simple Summary

Intervertebral Disc Disease in dogs frequently requires surgical decompression through hemilaminectomy. The present study aimed to evaluate postoperative changes in local cutaneous temperature and thermal patterns in dogs with thoracolumbar intervertebral disc extrusion using Infrared Thermography. Fifteen dogs diagnosed with Hansen type I thoracolumbar intervertebral disc extrusion were included. Thermographic evaluation of the thoracolumbar region (T11–L3) was performed preoperatively (Day 0), 24 h postoperatively (Day 1), and 7 days after surgery (Day 7) using an FLIR E50 thermal camera. Mean cutaneous temperature and thermal distribution patterns were analyzed. Statistical analyses were conducted using Repeated Measures Analysis of Variance (ANOVA) and the Friedman non-parametric test. Significant differences in local cutaneous temperature were identified between the evaluated time points (*p* < 0.001). Thermographic assessment demonstrated progressive modifications in thermal distribution throughout the postoperative period. No postoperative complications, including seroma formation, wound dehiscence, or fistula development, were observed during the monitoring period. These findings suggest that infrared thermography may represent a useful complementary non-invasive method for postoperative monitoring of tissue healing following hemilaminectomy in dogs.

## 1. Introduction

Intervertebral Disc Disease (IVDD) is a common neurological disorder in dogs and occurs most frequently in chondrodystrophic breeds due to their predisposition to intervertebral disc degeneration [1,2,3]. The condition is characterized by degeneration and herniation of intervertebral disc material, resulting in spinal cord compression of varying severity [4]. The thoracolumbar region, especially the T12–L3 spinal segments, is most frequently affected, with clinical signs ranging from spinal pain to paraparesis or paraplegia [5,6].

Diagnosis is based on neurological examination and advanced imaging techniques. Neurological assessment enables lesion localization and evaluation of disease severity, while computed tomography and magnetic resonance imaging provide confirmation of disc extrusion and support surgical planning [7,8,9]. Neurological assessment allows lesion localization and evaluation of neurological severity, while magnetic resonance imaging (MRI) is considered the gold standard for definitive diagnosis and accurate assessment of spinal cord compression [10,11]. Computed tomography (CT), myelography, or a combination of these modalities may also be used depending on clinical availability [12,13,14].

Hemilaminectomy is commonly performed in dogs presenting with significant neurological deficits or failure of conservative treatment [15,16]. Although postoperative outcomes are generally favourable, neurological recovery is influenced by factors such as preoperative neurological status, timing of surgical intervention, and preservation of deep pain perception [17,18].

Beyond neurological recovery, postoperative tissue healing, particularly cutaneous wound healing, represents an important aspect of clinical outcome assessment [19,20]. Conventional evaluation of surgical wound healing is primarily based on subjective clinical observation of local signs such as erythema, swelling, heat, and pain, which may vary depending on the examiner’s experience and clinical interpretation [19,21].

In this context, non-invasive imaging modalities have gained increasing interest for the objective assessment of inflammation and local tissue healing [19,22,23]. Infrared Thermography enables the detection of cutaneous temperature variations associated with changes in local blood perfusion and inflammatory response [24,25]. This technique provides an objective, repeatable, and non-invasive method for evaluating tissue thermal dynamics, which may indirectly reflect different stages of postoperative healing [19,23,26,27].

The application of infrared thermography in postoperative veterinary patients offers the potential to monitor inflammatory evolution and detect early deviations from normal wound healing processes [28,29,30]. In cases of thoracolumbar intervertebral disc surgery, serial thermal assessment of the surgical site may provide valuable additional information regarding local tissue response following surgical intervention [28,29,30].

The objectives of this study were as follows:

- To perform a qualitative analysis of the thermal pattern in dogs affected by Intervertebral Disc Disease undergoing surgical decompression;
- To compare local cutaneous temperature in the thoracolumbar region (T11–L3) across three time points: preoperatively (Day 0), 24 h postoperatively (Day 1), and 7 days postoperatively (Day 7), using Infrared Thermography.

The study further aimed to evaluate whether Infrared Thermography can detect significant temporal changes in local temperature that may reflect postoperative inflammatory response and tissue healing dynamics in the early recovery period.

The null hypothesis of the present study was that no significant temporal changes in local cutaneous temperature or thermal patterns of the thoracolumbar region would be detected in dogs undergoing postoperative recovery following surgical treatment for Intervertebral Disc Disease in dogs, as assessed by Infrared Thermography.

## 2. Materials and Methods

### 2.1. Study Animals and Clinical Assessment

Cases included in this study were evaluated at the Surgery Clinic of the Faculty of Veterinary Medicine, Timisoara, between May 2022 and April 2024. During the study period, 23 dogs presenting with acute paraplegia and clinical suspicion of Intervertebral Disc Disease (IVDD) were assessed. The animals ranged in age from 4 years and 9 months to 7 years and 4 months and had body weights between 4.2 and 24 kg.

The inclusion criteria established for this study comprised dogs diagnosed with Hansen type I disc extrusion localized within the thoracolumbar spinal segments T11–L3, presented for neurological evaluation within 12 h after the onset of paraplegia, reduced or absent proprioception, or paraplegia with intact deep pain perception, with a rectal temperature ranging between 38 °C and 39.5 °C.

Dogs were excluded from the study if their body weight exceeded 20 kg, if the intervertebral disc extrusion was located outside the T11–L3 spinal segments, or if more than one disc extrusion was identified within the evaluated thoracolumbar region. Additional exclusion criteria comprised the presence of spinal disorders such as spondylosis involving the T11–L3 segments, discospondylitis, hemivertebrae, neoplastic lesions, and thromboembolic conditions. Cases previously treated with anti-inflammatory medication at other veterinary facilities or private practices were also omitted from the study, together with dogs exhibiting urinary and/or fecal incontinence.

Each patient was subjected to a detailed neurological assessment conducted by clinicians experienced in spinal cord disorders. The examination protocol included evaluation of superficial and deep pain sensation, panniculus response, and patellar and anal reflexes, as well as proprioceptive function [5]. Following neurological assessment, neurological status was graded for each dog using a modified scoring system adapted from the classification described by Wheeler and Sharp [31], where grade 1 consisted of spinal pain without neurological deficits, grade 2 of ambulatory paraparesis and proprioceptive ataxia, grade 3 of non-ambulatory paraparesis, grade 4 of paraplegia with preserved deep pain perception, and grade 5 of paraplegia with absence of deep pain perception [31]. Based on the neurological deficits identified during examination, the lesion was localized to the thoracolumbar segments of the spinal cord.

### 2.2. Imaging Evaluation

Diagnostic confirmation was achieved through computed tomography (CT) examination performed with a Siemens Somatom Definition AS 64 scanner (Siemens Healthineers, Erlangen, Germany), using both native and contrast-enhanced acquisitions. Prior to imaging, general anesthesia was induced with dexmedetomidine (Dexdomitor 0.5 mg/mL, Zoetis, Bucharest, Romania) at a dose of 10 mcg/kg intravenously and butorphanol (Butomidor 10 mg/mL, VetViva Richter GmbH, Bucharest, Romania) at a dose of 0.2 mg/kg body weight intravenously, while propofol was administered intravenously at a dose of 4 mg/kg (Propofol 10 mg/mL, Braun, Sânandrei, Romania) when necessary to maintain an adequate anesthetic depth. During scanning, dogs were positioned in dorsal recumbency. Image reconstruction was carried out using multiplanar reconstruction (MPR) techniques with a slice thickness of 1 mm.

**Figure 1:**
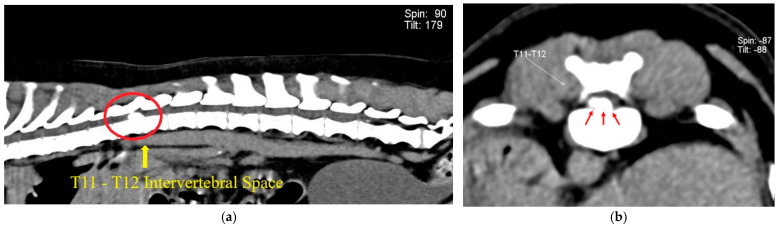
Intervertebral disc extrusion at the T11–T12 intervertebral space: (**a**) sagittally reconstructed view of the vertebral column, red circle—extruded disc material, yellow arrow—intervertebral space T11–T12; (**b**) red arrows—extruded disc material.

**Figure 2:**
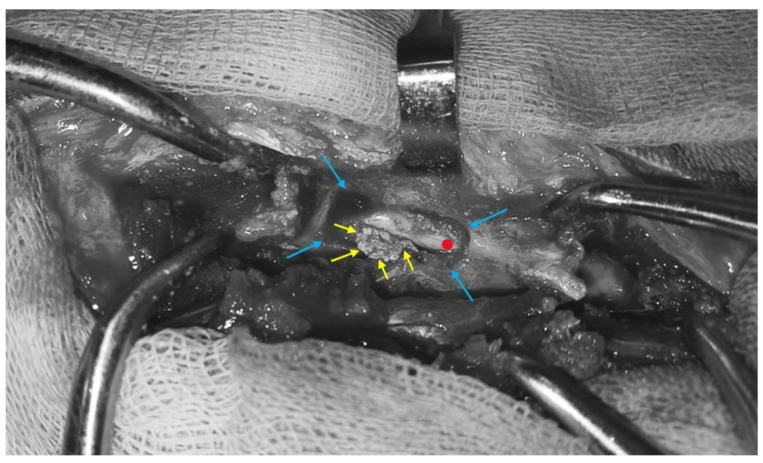
Intraoperative image during hemilaminectomy: blue arrows indicate the burred vertebral margins, yellow arrows indicate the extruded disc material, and the red dot indicates the spinal cord.

Based on the CT findings (Figure 1), all selected dogs were diagnosed with Hansen type I intervertebral disc extrusion. Surgical management consisted of hemilaminectomy performed by the same surgical team, with the surgical side selected according to the localization of the disc extrusion. For the hemilaminectomy procedure, a dorsal midline incision was performed over the affected vertebral segment, followed by dissection and lateral retraction of the longissimus muscle (*Longissimus dorsi*) and multifidus muscle (*Musculus multifidus*) to expose the left or right vertebral lamina and articular facets [15]. A left- or right-side hemilaminectomy was created using a high-speed burr, allowing access to the vertebral canal [15,32]. Following exposure of the vertebral canal, the extruded disc material responsible for spinal cord compression was located and gently extracted using fine surgical instrumentation (Figure 2). Following decompression, the surgical site was lavaged with sterile saline solution. In all dogs, closure was performed using a standardized protocol. The thoracolumbar fascia was sutured with absorbable suture material in a simple continuous pattern using Polydioxanone 2-0, followed by closure of the subcutaneous tissue using the same material. Skin closure was achieved using a continuous intradermal suture pattern with the absorbable monofilament material Polydioxanone 3-0. No skin staples or external skin sutures were used [15,32].

Postoperative analgesic management consisted of methadone administered at a dose of 0.2 mg/kg every 8 h during the first 24 h following surgery (8, 16, and 24 h postoperatively). Additional analgesia was provided with pregabalin administered orally at a dose of 4 mg/kg every 8 h for 7 consecutive days. Anti-inflammatory therapy consisted of meloxicam administered subcutaneously at a dose of 0.1 mg/kg once daily for 5 days following surgery. The same postoperative analgesic and anti-inflammatory protocol was applied to all dogs included in the study.

### 2.3. Thermographic Evaluation and Data Acquisition

Cutaneous temperature was evaluated by means of infrared thermography in order to identify local thermal variations associated with the postoperative healing process. Thermographic assessments were performed at three different time points for each patient: prior to surgery (Day 0), 24 h after the surgical procedure (Day 1), and 7 days postoperatively (Day 7).

**Figure 3:**
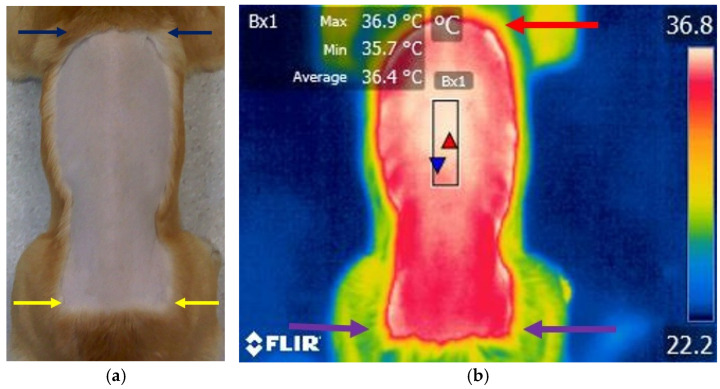
Image of the thoracolumbar area of a paraplegic dog: (**a**) Image with FLIR E50 camera—normal view, (**b**) image with Flir E50 camera—thermographic view. Arrows: (**a**) blue arrows—T5 level, yellow arrows—S1 level; (**b**) red arrows—T5 level, purple arrows—S1 level.

Before image acquisition, the hair covering the thoracolumbar region was clipped over an area extending approximately 5 cm around the surgical field, depending on the anatomical localization of the lesion. To reduce potential imaging artifacts caused by hair regrowth, crust formation, or local secretions, clipping was repeated on postoperative Day 4 (Figure 3).

All thermographic examinations were carried out under standardized environmental conditions, maintaining a constant ambient temperature of approximately 21 °C, relative humidity close to 70%, and elimination of air currents within the examination room. Prior to thermal image acquisition, each dog underwent an acclimatization period of approximately 2 h to allow stabilization of cutaneous temperature.

Thermal images were acquired using an FLIR E50 infrared thermal camera (FLIR Systems Inc., Wilsonville, OR, USA) with an emissivity setting of 0.95 and an image resolution of 240 × 180 pixels. The device was configured to measure temperatures within a range of −20 °C to 650 °C, with a thermal sensitivity of ≤0.05 °C. For all examinations, the thermal camera was positioned perpendicular to the thoracolumbar region at a distance of 1 m from the patient to ensure consistency and reproducibility of image acquisition. A standardized region of interest ROI—Bx1 measuring 98 × 60 pixels was established over the thoracolumbar area, centred on the surgical site within the T11–L3 segment. The mean surface temperature obtained from this region was used for further analysis (Figure 3).

### 2.4. Statistical Analysis

Local cutaneous temperature measurements were obtained from a cohort of N = 15 dogs at three distinct time points: preoperatively (Day 0), 24 h following surgery (Day 1), and 7 days postoperatively (Day 7). Repeated assessments of the same subjects were performed to investigate potential temporal changes in temperature throughout the postoperative recovery period.

Normality of data distribution was assessed using the Shapiro–Wilk test. Comparisons between the three time points were performed using Repeated Measures ANOVA implemented in JASP software (version 0.17.3). In addition, the non-parametric Friedman test was applied to validate the obtained results.

For thermographic analysis, mean local temperature values recorded within the region of interest were used for statistical evaluation.

## 3. Results

### 3.1. Study Population

A total of 23 dogs diagnosed with thoracolumbar Intervertebral Disc Disease were initially assessed during the study period. A total of 15 dogs fulfilled the inclusion criteria and were included in the study, whereas 8 dogs were excluded.

The study population consisted of dogs aged between 5.2 and 7.8 years (mean age: 5.8 years), with a mean weight of 9.25 kg (range: 3.80–19.90 kg). The study included the following breeds: four French Bulldogs, two Bichon Frises, two Dachshunds, three Shih Tzus, two Chihuahuas and two mixed breeds.

Exclusion criteria comprised absence of deep pain perception (n = 4), duration of clinical signs exceeding 12 h prior to presentation (n = 2), and the presence of discospondylitis involving the region of interest (n = 2).

Following clinical and neurological evaluation, five dogs were assigned to neurological grade 1, three dogs to grade 2, three dogs to grade 3, and four dogs to grade 4. Neurological grading was established according to a modified classification system adapted from the criteria described by Wheeler and Sharp [31].

Throughout the 7-day postoperative evaluation period, no evidence of seroma formation, erythema, or wound dehiscence was observed in any of the examined dogs. Rectal temperature was recorded at the beginning of the clinical examination and ranged between 38.0 °C and 39.5 °C in all evaluated dogs.

### 3.2. Advanced Imaging Evaluation

A total of 15 computed tomography examinations demonstrating intervertebral disc extrusion were included in the study. Lesion localization was distributed as follows: two cases at the T11–T12 intervertebral space, two cases at T12–T13, five cases at T13–L1, three cases at L1–L2, and three cases at L2–L3. Regarding the position of the extruded disc material within the vertebral canal, ventral localization beneath the spinal cord was identified in five cases, left lateralization in six cases, and right-sided localization in four cases.

### 3.3. Infrared Thermographic Assessment

Day 0—Preoperative Assessment

**Figure 4:**
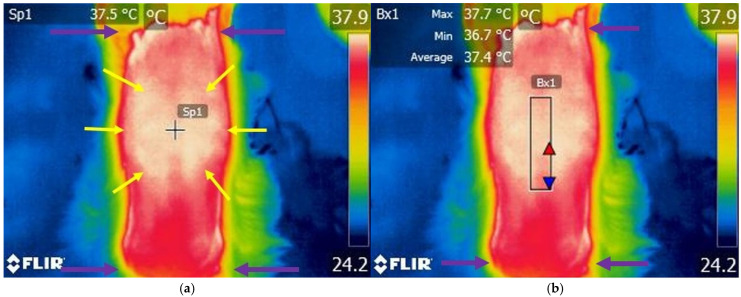
Representative thermographic image of the thoracolumbar region in a dog diagnosed with T12–T13 intervertebral disc extrusion. (**a**) Thermal image obtained prior to software processing: the purple arrow indicates the clipped area extending from T5 to S1, the yellow arrow highlights a region of increased temperature, and Sp1 denotes the local skin temperature measurement point. (**b**) Thermographic image analyzed using FLIR Tools software 5.X.: the purple arrows identifies the clipped area between T5 and S1, Bx1 represents the region of interest (ROI) centred over the surgical site, the red triangle indicates the highest temperature recorded within the ROI, and the blue triangle indicates the lowest temperature recorded within the same area.

Preoperative thermographic evaluation of the thoracolumbar region demonstrated a localized area of increased cutaneous temperature extending between the T11 and L3 spinal segments (Figure 4). Within the defined region of interest (ROI), the mean recorded cutaneous temperature was 36.16 °C (Table S1).

Day 1—24-Hour Postoperative Assessment

**Figure 5:**
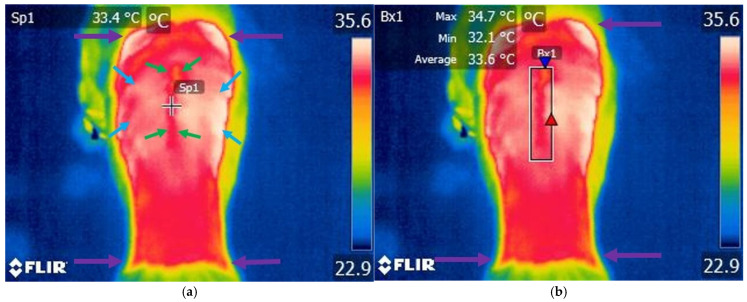
Representative thermographic image of the thoracolumbar region in a dog affected by T12–T13 intervertebral disc extrusion. (**a**) Thermal image obtained prior to FLIR Tools analysis: purple arrows indicate the clipped area extending from T5 to S1, the green arrow identifies a region of lower temperature, the blue arrows highlight areas of increased temperature, and Sp1 denotes the local skin temperature measurement point. (**b**) Thermographic image processed using FLIR Tools software: purple arrows indicate the clipped area between T5 and S1, Bx1 represents the region of interest (ROI) centred over the surgical site, the red triangle marks the highest temperature recorded within the ROI, and the blue triangle marks the lowest temperature recorded within the same region.

Thermographic evaluation performed 24 h following hemilaminectomy revealed a distinct thermal distribution characterized by a centrally localized area of reduced cutaneous temperature corresponding to the surgical incision line and extending dorsally along the vertebral region. This hypothermic area was bilaterally surrounded by regions of increased cutaneous temperature adjacent to the incision site (Figure 5). Within the defined region of interest (ROI), the mean cutaneous temperature recorded was 34.24 °C (Table S1).

Day 7—Seven-Day Postoperative Assessment

**Figure 6:**
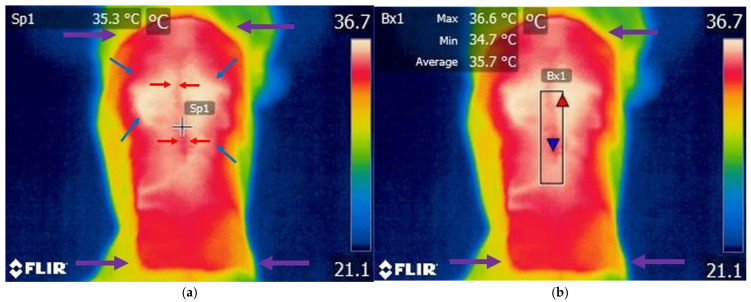
Representative thermographic image of the thoracolumbar region in a dog diagnosed with T12–T13 intervertebral disc extrusion. (**a**) Thermal image obtained prior to FLIR Tools analysis: purple arrows indicate the clipped area extending from T5 to S1, red arrows identify regions of reduced temperature, blue arrows highlight areas of increased temperature, and Sp1 denotes the local skin temperature measurement point. (**b**) Thermographic image processed using FLIR Tools software: the purple arrows indicates the clipped area between T5 and S1, Bx1 represents the region of interest (ROI) centred over the surgical site, the red triangle marks the highest temperature recorded within the ROI, and the blue triangle marks the lowest temperature recorded within the same region.

Thermographic evaluation performed seven days following hemilaminectomy demonstrated a discrete area of reduced temperature that remained identifiable along the surgical incision line, surrounded by regions of increased cutaneous temperature adjacent to the surgical site (Figure 6). Within the defined region of interest (ROI), the mean cutaneous temperature recorded was 34.84 °C (Table S1).

### 3.4. Group Comparison

**Figure 7:**
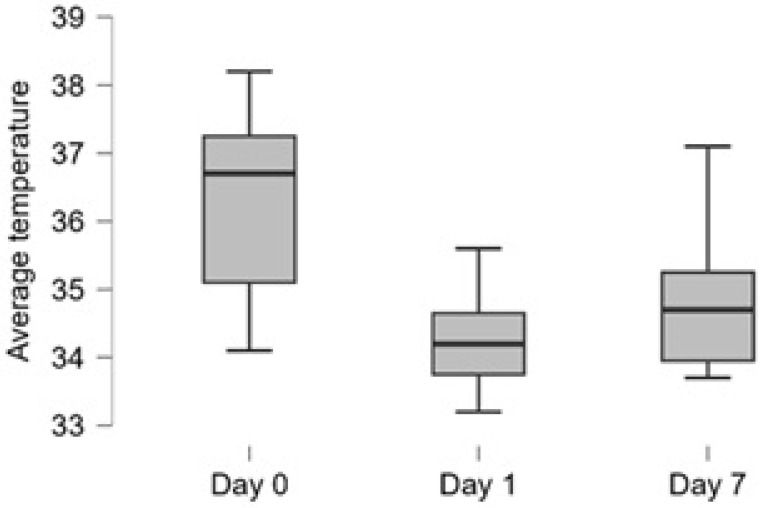
Boxplot representation of the mean temperature distributions recorded at the three evaluated time points.

Application of the Repeated Measures ANOVA revealed a significant effect of time on local cutaneous temperature measurements, indicating that mean temperature values differed significantly among the three evaluated time points in relation to the surgical intervention (*p* < 0.001) (Figure 7). The calculated effect size was high (η^2^ = 0.75), demonstrating a strong influence of postoperative time on thermal variation. To further assess the robustness of the statistical findings, the non-parametric Friedman test was additionally performed, yielding consistent results and confirming the presence of significant differences between the evaluated measurement series (Table S2).

Post hoc pairwise comparisons were subsequently performed. Bonferroni-adjusted *p*-values revealed significant differences between preoperative mean temperature values and both postoperative time points (*p* < 0.001), as shown in Table 1. Furthermore, a statistically significant difference was also identified between the two postoperative evaluations, indicating a continued change in mean temperature over time (*p* = 0.024), as shown in Table 1.

## 4. Discussion

The thermographic evaluation of the thoracolumbar region provided relevant information regarding the temporal evolution of local cutaneous temperature following hemilaminectomy in dogs affected by Intervertebral Disc Disease. The initial null hypothesis (H0), stating that no significant temperature differences would be identified between the three evaluated postoperative time points, was not supported by the obtained results. Application of the Repeated Measures ANOVA demonstrated a significant effect of time on local cutaneous temperature measurements, indicating the presence of significant thermal variations throughout the postoperative period (*p* < 0.001). In addition to quantitative differences in temperature values, thermographic assessment also revealed changes in thermal distribution patterns within the thoracolumbar region.

Preoperative thermographic evaluation (Day 0) revealed increased local cutaneous temperature in the thoracolumbar region. This finding may be attributed to the localized inflammatory response associated with disc extrusion, as well as secondary muscular contractions [33]. Muscle spasms are considered a consequence of nociceptive stimulation induced by pain, contributing to increased metabolic activity and regional heat production [34]. At 24 h postoperatively (Day 1), a statistically significant decrease in local temperature was observed compared to baseline values. The reduction in mean cutaneous temperature observed following surgical intervention may be associated with tissue trauma and disruption of local vascular structures, resulting in transient microcirculatory alterations [29,35]. By postoperative Day 7, an increase in mean cutaneous temperature was observed compared with Day 1 values. This thermal evolution may reflect progressive restoration of local microvascular perfusion and normalization of vascular dynamics associated with advancing tissue repair and modulation of the postoperative inflammatory response [29,35,36]. The identified thermal variations reflect the dynamic physiological processes associated with postoperative tissue response, including inflammatory changes, local vascular alterations, and progression of wound healing [29,35,37].

Prior to thermographic assessment, the hair over the region of interest was clipped, and all dogs were allowed an acclimatization period of approximately 2 h before image acquisition. This approach is consistent with previously published protocols, which recommend sufficient acclimatization time to ensure stabilization of cutaneous temperature and minimize external influences on thermal measurements [38,39]. In addition, environmental conditions were rigorously standardized, with ambient temperature maintained at approximately 21 °C throughout the acclimatization and thermographic acquisition procedures. An ambient temperature of approximately 21 °C has previously been reported as having minimal influence on cutaneous temperature and thermoregulatory mechanisms in animals [40].

This temperature range has been reported to minimize variability in cutaneous surface temperature measurements, thereby enhancing the reliability, consistency, and reproducibility of thermographic data [28,41].

In the present study, diagnosis was established through the combined use of neurological examination and computed tomographic evaluation, enabling accurate localization of the spinal lesion and confirmation of intervertebral disc extrusion. Similar diagnostic protocols have been extensively described in the veterinary literature, with several authors emphasizing the value of integrating neurological findings with advanced imaging modalities to improve diagnostic accuracy and facilitate surgical planning in dogs affected by Intervertebral Disc Disease [11,14,42].

In the present study, the T11–L3 spinal region was selected as the area of interest due to the high frequency of intervertebral disc extrusions reported at this level. Previous studies by Wheeler and Sharp [31] indicated that more than 50% of thoracolumbar disc extrusions occur at the T12–T13 and T13–L1 intervertebral spaces, while over 85% are localized between T11–T12 and L2–L3 [43].

Surgical management in the present study consisted of left- or right-sided hemilaminectomy centred over the intervertebral space affected by disc extrusion, with the surgical approach selected according to the localization of the extruded disc material identified on computed tomographic examination. Throughout the postoperative monitoring period, no local complications, including seroma formation, wound dehiscence, or fistula development, were identified in any of the included dogs. Similar findings have been reported by other authors, who describe hemilaminectomy as a reliable and well-tolerated surgical technique for thoracolumbar Intervertebral Disc Disease in dogs when appropriate surgical planning and postoperative management are applied [32,44].

Postoperative analgesic and anti-inflammatory management may influence local inflammatory activity, vascular dynamics, and microvascular perfusion, factors that are closely associated with cutaneous temperature distribution. Meloxicam, through inhibition of cyclooxygenase activity, may contribute to modulation of inflammatory responses and local vascular changes during the postoperative period [45]. Similarly, adequate analgesia may reduce neurogenic and stress-related vascular responses [46]. However, as all dogs were treated according to the same postoperative protocol, the influence of pharmacological therapy was considered consistent throughout the study population and, therefore, unlikely to affect comparisons between the evaluated time points.

Further investigations are warranted to evaluate the relationship between thermographic findings and postoperative neurological recovery. In particular, future studies should compare local temperature patterns between dogs exhibiting favourable neurological recovery and those presenting persistent paraplegia or residual neurological deficits, in order to determine whether thermographic variations may correlate with clinical outcome and functional recovery following spinal surgery.

Several limitations related to both the surgical and thermographic procedures should be acknowledged. Variability among the included dogs, including differences in breed, body conformation, skin pigmentation, thoracolumbar musculature, and distribution of subcutaneous adipose tissue, may have influenced local thermal measurements and thermographic pattern interpretation. Variations in the extent of muscular dissection and soft tissue manipulation during hemilaminectomy may have influenced local vascular perfusion and postoperative thermal distribution within the thoracolumbar region. Another limitation of the present study was the use of computed tomography for diagnostic evaluation, which does not allow assessment of intramedullary spinal cord alterations, such as edema, hemorrhage, or ischemic changes. Furthermore, thermographic assessment remains sensitive to multiple technical and physiological factors, including positioning of the patient, angle and distance of image acquisition, environmental conditions, and individual anatomical variability, all of which may influence the accuracy and reproducibility of temperature measurements. In addition, slight variations in the localization of the disc extrusion and hemilaminectomy site within the T11–L3 region may have influenced the observed thermal patterns.

## 5. Conclusions

Infrared thermography enabled the identification of significant temporal variations in local cutaneous temperature following hemilaminectomy in dogs with thoracolumbar intervertebral disc extrusion.

The thermographic changes observed throughout the postoperative period likely reflect dynamic physiological processes associated with inflammation and local microvascular perfusion.

These findings suggest that infrared thermography may represent a useful complementary non-invasive tool for postoperative monitoring. Further studies are required to determine its potential role in assessing wound healing and predicting clinical outcomes.

## Figures and Tables

**Table 1:** Post hoc comparisons of mean local temperature values recorded in dogs across the three evaluated time points.

Interval Time Comparison	Mean Difference	SE	t	Cohen’s d	*p*—Bonferroni
Day 0–Day 1	1.913	0.210	9.119	1.804	<0.001
Day 0–Day 7	1.313	0.210	6.259	1.238	<0.001
Day 1–Day 7	−0.600	0.210	−2.860	−0.566	0.024

## Data Availability

The data supporting the findings of the present study are available within the article and its Supplementary Materials. Additional information may be obtained from the corresponding author.

## Supplementary Materials

The following supporting information can be downloaded at: https://www.mdpi.com/article/10.3390/ani16121796/s1, Table S1: Statistical summary of mean local temperatures recorded at the three evaluated time points; Table S2: Non-parametric Friedman results.
