# Supplementary material for: Infrared Thermographic Evaluation Following Hemilaminectomy in Dogs with Thoracolumbar Intervertebral Disc Extrusion: A Pilot Study
Source: Animals (Basel). 2026 Jun 10;16(12):1796. doi: 10.3390/ani16121796 (PMC13296032; doi:10.3390/ani16121796)
Supplement: Supplementary file 1 [file animals-16-01796-s001.zip › Table S1. Statistical summary of mean local temperatures recorded at the three evaluated time points.pdf]

Statistical summary of mean local temperature values recorded in the study population of dogs at the three evaluated time points

	**Day 0**	**Day 1**	**Day 7**
N	15	15	15
Median	36.700	34.200	34.700
Mean	36.160	34.247	34.847
Std. Deviation	1.332	0.712	1.045
Shapiro-Wilk	0.915	0.959	0.907
P-value of Shapiro-Wilk	0.161	0.681	0.120
Minimum	34.100	33.200	33.700
Maximum	38.200	35.600	37.100
Interquartile range	2.15	0.9	1.3
