# Supplementary material for: Infrared Thermographic Evaluation Following Hemilaminectomy in Dogs with Thoracolumbar Intervertebral Disc Extrusion: A Pilot Study
Source: Animals (Basel). 2026 Jun 10;16(12):1796. doi: 10.3390/ani16121796 (PMC13296032; doi:10.3390/ani16121796)
Supplement: Supplementary file 1 [file animals-16-01796-s001.zip › Table S2. Non-parametric Friedman test results.pdf]

Nonparametrics test

Friedman Test (average temperature)

Factor	Chi-Squared	df	p	Kendall's W
RM Factor 1	28.133	2	< .001	0.938
